# Transcriptomic insights into common defense mechanisms of *Pleurotus ostreatus*, *Agaricus bisporus*, and *Lentinula edodes* against *Pseudomonas tolaasii*-induced browning

**DOI:** 10.3389/ffunb.2026.1737784

**Published:** 2026-03-04

**Authors:** Min-Seo Jung, Ha-Young Pyeon, Youn-Jin Park, Myoung-Jun Jang

**Affiliations:** 1 Laboratory of Applied Biology, Department of Plant Resources, College of Industrial Sciences, Kongju National University, Kongju, Republic of Korea; 2 Technology Institute, KBNP Inc., Yesan, Republic of Korea

**Keywords:** *Agaricus bisporus*, defense response, laccase, *Lentinula edodes*, *Pleurotus ostreatus*, *Pseudomonas tolaasii*

## Abstract

This study investigated gene expression patterns associated with the browning of mushroom fruiting bodies caused by *Pseudomonas tolaasii* infection. To address this, *Pleurotus ostreatus, Agaricus bisporus*, and *Lentinula edodes* were inoculated with *P. tolaasii*, and morphological changes were examined, followed by transcriptome analysis to identify common and differentially expressed genes across the three species. Hydrophobin and glycoside hydrolase family 13 genes, associated with α-glucan metabolism and cell wall-related processes, were commonly downregulated, whereas laccase- and histidine kinase-related genes involved in melanin biosynthesis were significantly upregulated (log2 fold change ≥ 2, p ≤ 0.05). Notably, laccase induction represented a conserved transcriptional response to *P. tolaasii* infection, although the magnitude of induction varied among species. These findings provide molecular evidence linking mushroom browning to the transcriptional activation of defense-related genes and highlight laccase as a key, yet species-dependent, component of the response to *P. tolaasii* infection.

## 1 Introduction

Mushrooms are a group of multicellular fungi characterized by the formation of macroscopic fruiting bodies for spore production and predominantly belong to the phyla Basidiomycota and Ascomycota (Choi et al., 2010).

Edible mushrooms contain essential amino acids when evaluated on a dry matter basis, and various species, including *Agaricus bisporus*, *Pleurotus ostreatus*, and *Lentinula edodes*, are cultivated worldwide (Korea Forest Service, 2019). *A. bisporus* contains protein and vitamins and exhibits anticancer-related bioactivity (Barros et al., 2008). *P. ostreatus* shows immune-enhancing and antibacterial activities (Bellettini et al., 2019), whereas *L. edodes* contains bioactive compounds such as lentinan and eritadenine and is known for its pharmacological properties (Wasserman, 2005; Moon et al., 2015).

Filamentous fungi are susceptible to various diseases during cultivation, with bacterial brown blotches being a significant affliction that causes considerable damage (Lee, 1999). This disease is attributed to bacteria of the genus *Pseudomonas*, particularly *Pseudomonas tolaasii* (Moallem et al., 2024), and affects several edible mushroom species, including *A. bisporus*, *P. ostreatus*, and *L. edodes* (Basim and Basim, 2018). The pathogen produces an extracellular toxin known as tolaasin, which disrupts the cell membranes of mushroom cells and enhances tyrosinase activity in the presence of oxygen, resulting in browning symptoms in the fruiting bodies (Yun et al., 2023; Bassarello et al., 2004).

The browning reaction is facilitated by polyphenol oxidase (PPO), an enzyme that not only catalyzes enzymatic browning but also plays a crucial role in plant defense against both biotic and abiotic stresses (Molitor et al., 2016; Pourcel et al., 2007). In response to pathogen and pest attacks, plants increase the expression of PPO genes as a natural defense mechanism. For instance, the JrPPO1 gene in *Juglans regia* was upregulated in response to bacterial infections, indicating that PPO was integral to the plant’s defense strategy (Khodadadi et al., 2016). When plant tissues were damaged, signaling molecules such as salicylic acid and jasmonates stimulated the expression of PPO genes.

PPO localizes in cellular organelles and induces browning when the cell membrane is damaged by wounding or pathogen attack, allowing it to come into contact with phenolic compounds stored in the vacuole (Zhang, 2023; Guo et al., 2025). In mushrooms, browning also functions as a defense response, in which quinones produced by PPO are polymerized into melanin to protect the tissue (Liang et al., 2024).

Depending on substrate specificity and reaction context, PPO-related activities include enzymes such as tyrosinases, catechol oxidases, and multicopper oxidases, including laccases (Mishra and Gautam, 2016; Pretzler and Rompel, 2018). PPO-mediated reactions generally involve two key steps: the hydroxylation of monophenols to o-diphenols and the subsequent oxidation of o-diphenols to o-quinones (Tomás‐Barberán and Espín, 2001). These reactive o-quinones can further undergo non-enzymatic polymerization or condense with amino acids and proteins, leading to the formation of brown pigments characteristic of browning responses (Ramaswamy and Riahi, 2003; Torres et al., 2021). In this context, laccase (EC 1.10.3.2), a member of the multicopper oxidase family, catalyzes the oxidation of a wide range of aromatic compounds while reducing molecular oxygen to water (Egbewale et al., 2024; Orlando et al., 2024). Fungal laccase can oxidize not only phenolic compounds but also aromatic amines, polycyclic aromatic hydrocarbons, synthetic dyes, antibiotics, and non-phenolic substrates. This versatility is closely related to both simple metabolic processes and environmental adaptation strategies for survival (Mikolasch and Schauer, 2009; Polak and Jarosz-Wilkolazka, 2012). Thus, the multifunctionality and enzymatic activity of laccase suggest that it plays a crucial role in the defense mechanisms by which mushrooms respond to various environmental stresses and pathogen attacks.

Laccase expression in *P. ostreatus* is upregulated during co-cultivation with various *Trichoderma* strains, particularly *T. viride* CIAD 01 and *T. lignorum* CIAD 02. In contrast, *A. bisporus* exhibits higher basal laccase activity in monoculture; however, it shows only moderate increases upon co-cultivation with strains such as *T. harzianum* CIAD 05 and *Trichoderma* sp (Flores et al., 2009).

Similarly, in *L. edodes* co-cultured with *Trichoderma*, an increase in laccase activity is observed in response to antagonistic interactions. A brown line forms at the contact area between *L. edodes* mycelia and *Trichoderma*, which is thought to serve as a defensive barrier, creating a boundary against the pathogen (Savoie and Mata, 1999). This indicates that mushrooms can activate their defense mechanisms by modulating laccase expression in response to pathogen attacks, providing important insights into the disease resistance mechanisms of mushrooms. Previous transcriptomic and metabolomic analyses have investigated the defense responses of *A. bisporus* to *P. tolaasii* infection, highlighting the involvement of pathogenesis-related (PR) proteins, reactive oxygen species (ROS)-related genes, and secondary metabolite pathways (Yang et al., 2022). However, comparative studies among different mushroom species remain limited.

In this study, *A. bisporus*, *P. ostreatus*, and *L. edodes* were artificially inoculated with the pathogen *P. tolaasii* to investigate transcriptional responses associated with bacterial brown blotch disease. This study aimed to examine whether *P. tolaasii* infection induces common defense-associated responses across different edible mushroom species, with a particular focus on browning-related pathways linked to phenolic compound oxidation. To address this, commonly expressed genes were identified through transcriptome analysis, and representative laccase genes were selected as indicators of browning-related defense responses to better understand conserved defense mechanisms among these edible mushroom species.

## 2 Materials and methods

### 2.1 Strain cultivation and growth conditions

The pathogenic bacterium *P. tolaasii* (KACC 10038), utilized in this study, was obtained from the Korean Agricultural Culture Collection. The bacterial strain was first cultured on LB agar medium at 28°C for 24 hours and then inoculated into 250 mL of LB liquid medium in a 500 mL flask. The culture was subsequently incubated at the same temperature with shaking at 120 rpm.

*P. ostreatus* ‘Heuktari’ (ASI0665) was obtained from the Environmental Microbiology Research Institute of the Gyeonggi Agricultural Technology Institute and was cultured on PDA medium at 25°C for 7 days. Subsequently, the strain was inoculated into a substrate composed of 80% poplar sawdust and 20% rice bran (v/v) and was incubated for 30 days to produce spawn. For mycelial growth, the strain was cultured on a substrate consisting of 50% poplar sawdust, 30% beet pulp, and 20% cottonseed meal (v/v), with a moisture content adjusted to 65%, and was incubated at 23°C for 30 days. Fruiting body formation was induced at 20°C, with 95% relative humidity and a CO_2_ concentration of 3,000 ppm. The mature fruiting bodies were harvested after 8 to 11 days of growth, when the pileus reached approximately 3 cm in diameter.

*A. bisporus* ‘Saehan’ (KPAB022821) was obtained from the Department of Plant Resources at Kongju National University and was cultured on CDA medium at 25°C for 14 days. For spawn production, a grain-based medium was prepared by mixing 20 kg of wheat, 0.6 kg of gypsum, and 0.3 kg of calcium carbonate per 100 bottles. The medium was inoculated and was incubated for 15 to 30 days to produce spawn. For bed cultivation, a fermented rice straw compost substrate was utilized. The substrate was fermented at 65°C for 6 hours and then was incubated at 55°C for approximately 7 days. It was subsequently placed into boxes (523 × 366 × 183 mm, 26 L) at a density of 10 kg per box, with 500 g of spawn inoculated into each. The substrate was incubated at 25°C with a humidity level of 65% for 15 days. Once mycelial colonization exceeded 80%, casing was applied. After 15 days of casing, fruiting was induced by adjusting the temperature to 16 ± 2°C and the humidity to 85%. The fruiting bodies were harvested before the pileus fully expanded.

*L. edodes* ‘Sanjo 701’ (ASI3305) was obtained from the Mushroom Division of the Rural Development Administration and was cultured on PDA medium at 25°C for 7 days. Subsequently, the strain was inoculated into a substrate composed of 80% oak sawdust and 20% rice bran (v/v) and was incubated at 25°C for 20 days. The same substrate was sterilized under high pressure, inoculated, and was incubated for 30 days to produce spawn. For cultivation, the substrate moisture content was adjusted to approximately 60%, and 1.2 kg of the substrate was packed into each bag. A 10–15 g portion of the spawn was inoculated into each bag. The incubation process involved dark incubation at 20 ± 1°C for 60 days, followed by light exposure for 30 days to induce browning.

### 2.2 Pathogen inoculation and infection symptom evaluation

Fruiting bodies for pathogen inoculation were selected based on the absence of browning or physical damage on the surfaces of the pileus and stipe. All experimental procedures were conducted in a clean bench to maintain sterile conditions. Each experiment was performed using three independent biological replicates, and to minimize transcriptomic variation due to treatment conditions, only morphologically uniform fruiting bodies at the same developmental stage were used. All inoculations and storage conditions were standardized accordingly.

The freeze-dried powder of the pathogen *Pseudomonas tolaasii* was rehydrated in 500 µL of sterile distilled water and inoculated onto LB agar (Luria-Bertani broth agar) medium. The plate was incubated at 28°C for 24 hours. The cultured bacteria were then transferred to LB liquid medium and incubated under the same conditions. Subsequently, 100 µL aliquots of the culture were spread on LB agar plates and incubated again at 28°C for 24 hours prior to use in the experiments.

Inoculation of *P. tolaasii* was performed using a colony plug method. To distinguish browning caused by pathogen activity from that caused by mechanical wounding or air exposure, a sterile needle was used to create a standardized 1 cm-deep cavity in the pileus tissue. The same needle puncture procedure was applied to both the negative control and pathogen-treated samples to control for the effect of mechanical injury. Sterile LB agar plugs without *P. tolaasii* were applied as a negative control, while LB agar plugs containing *P. tolaasii* were used as the pathogen treatment, to assess browning caused by the pathogen versus the medium alone.

The optical density (OD_600_) of the *P. tolaasii* culture used for inoculation was 0.238, and the colony-forming unit (CFU) concentration was measured as 2.8 × 10^6^ CFU/mL. Post-inoculation storage conditions were established based on previous findings that *P. tolaasii* rapidly proliferates and produces toxins under 16°C and 80–90% humidity, leading to disease symptoms on the pileus (Nair and Fahy, 1972).

The browning reaction on the surface of the pileus was visually and olfactorily assessed in comparison with the control. Browning first appeared in *A. bisporus* 24 hours after *P. tolaasii* inoculation, followed by *P. ostreatus* and *L. edodes*. In addition, cross-sections of browned fruiting bodies were observed using a stereomicroscope (SZ61TR, Olympus, Tokyo, Japan).

### 2.3 RNA extraction and quality assessment

Total RNA was extracted from the pileus surface of *P. ostreatus*, *A. bisporus*, and *L. edodes* fruiting bodies without browning (control group), as well as from the browned pileus of each species after artificial inoculation with *P. tolaasii* using a colony plug method.

RNA extraction was performed using the Ribospin™ II RNA Purification Kit (GeneAll^®^ Biotechnology, Seoul, Korea), with modifications to the manufacturer’s protocol as follows. Each fruiting body sample was ground in liquid nitrogen using a mortar and pestle. Then, 100 mg of the powdered sample was transferred into a sterile 2 mL microcentrifuge tube, followed by the addition of 700 µL of RNA lysis buffer provided in the kit. The mixture was vortexed for 30 seconds to ensure thorough mixing. After centrifugation at 10,000 × g for 2 minutes, 600 µL of the supernatant containing RNA was transferred to a new 1.5 mL microcentrifuge tube. An equal volume (600 µL) of 70% ethanol was added, and the solution was gently mixed by pipetting up and down. The mixture was then loaded onto a mini spin column and centrifuged at 10,000 × g for 1 minute. The flow-through was discarded. Next, 600 µL of RNA wash buffer was added to the column, and centrifugation was performed at 10,000 × g for 30 seconds. The flow-through was discarded again.

To remove genomic DNA contamination, 70 µL of DNase I reaction mixture (2 µL DNase I + 70 µL DRB buffer) was applied directly to the center of the membrane in the mini spin column, followed by incubation at room temperature for 10 minutes. RNA wash buffer (600 µL) was then added to the column and centrifuged at 10,000 × g for 30 seconds. This washing step was repeated once more.

To remove any residual wash buffer, the column was centrifuged at 14,000 × g for 2 minutes. The column was then placed into a new 1.5 mL microcentrifuge tube, and 50 µL of nuclease-free water was added to the center of the membrane. After incubating for 10 minutes at room temperature, RNA was eluted. This elution step was performed twice, and the collected eluates were pooled.

The quality of the extracted RNA was assessed using an Agilent TapeStation 4000 system (Agilent Technologies, Amstelveen, The Netherlands), and RNA concentration was determined using a spectrophotometer (Thermo Scientific, ND-2000, Madison, USA) (**Table 1**).

**Table 1:** RNA quality control result.

Sample		Concentration			Tapestation	
		ng/µL	Total (mg)	OD 260/280	Ratio (28s/18s)	RIN^a^
Control^*^	PO^b^	860.4	15.48	2.19	2.3	9.6
	AB^c^	409.9	7.37	2.33	2.0	10.0
	LE^d^	587.5	10.57	2.24	2.0	10.0
Treated	PO	860.4	7.88	2.26	2.4	9.8
	AB	409.9	11.48	2.25	1.1	8.0
	LE	587.5	8.76	2.30	2.3	10.0

^*^Control, Untreated mushroom; Treated, Treated mushroom (*P. tolaasii)*; ^a^RIN, RNA Integrity number;

^b^PO, *Pleurotus ostreatus*;

^c^AB, *Agaricus bisporus*;

^d^LE, *Lentinula edodes*.

To ensure consistency and comparability of gene expression results, the same total RNA samples extracted from the fruiting bodies were used for both RNA-Seq and qRT-PCR analyses.

### 2.4 QuantSeq 3’ mRNA-sequencing and data analysis

QuantSeq 3’ mRNA-sequencing was conducted to compare transcript expression between symptomatic and control fruiting bodies. RNA was extracted from *P. ostreatus*, *A. bisporus*, and *L. edodes* fruiting bodies and was subsequently used for sequencing, resulting in a total of six sequencing datasets.

The obtained sequencing data were filtered using BBduk (decontamination using kmers) to retain only reads with a quality score of Q20 or higher. Filtered QuantSeq 3′ mRNA-seq reads were aligned using Bowtie2 (Langmead and Salzberg, 2012), with Bowtie2 indices generated from either genome assembly sequences or representative transcript sequences for alignment at the genome and transcriptome levels. Specifically, the *Pleurotus ostreatus* genome assembly PleosPC15_2 (GCA_000697685.1; https://www.ncbi.nlm.nih.gov/datasets/genome/GCA_000697685.1/), the *Agaricus bisporus* reference genome Agabi_varbisH97_2 (GCA_021015755.1; https://www.ncbi.nlm.nih.gov/datasets/genome/GCF_000300575.1/), and the *Lentinula edodes* reference genome Lenedo1 (GCA_000300575.1; https://www.ncbi.nlm.nih.gov/datasets/genome/GCF_021015755.1/) were used for read alignment. The aligned reads were used to estimate gene expression levels, and read counts were obtained using Bedtools based on coverage from both unique and multiple alignments (Quinlan AR, 2010). Differentially expressed genes (DEGs) were identified using the edgeR package with TMM normalization and CPM-based filtering (Robinson et al., 2009).

Read count data were processed using Bioconductor (Gentleman et al., 2005) within R (R Core Team, 2021) and normalized using the TMM+CPM method with EdgeR. Gene classification was performed using the DAVID (http://david.abcc.ncifcrf.gov/) and the Medline database (http://www.ncbi.nlm.nih.gov/). Data mining and visualization were conducted using ExDEGA (Ebiogen Inc., Seoul, Korea).

The control and treated RNA samples used for sequencing were deposited in the NCBI Sequence Read Archive (SRA) under the BioProject accession number PRJNA1290832: SAMN49942090 (*P. ostreatus*, control), SAMN49942091 (*P. ostreatus*, treated), SAMN49942092 (*A. bisporus*, control), SAMN49942093 (*A. bisporus*, treated), SAMN49942094 (*L. edodes*, control), and SAMN49942095 (*L. edodes*, treated).

### 2.5 DEG analysis

For DEG analysis, completely untreated fruiting bodies (uninoculated control) were used as the reference group, allowing direct comparison of gene expression changes induced by *P. tolaasii* inoculation. Expression analysis for each gene following transcriptome analysis was conducted using ExDEGA (e-biogene, Seoul, Korea). Genes with increased or decreased expression were identified based on a threshold of 2-fold change. For the DEG scatter plot, genes exhibiting a 2-fold or greater change in expression (both up- and down-regulated) were analyzed, with the normalized RC (log_2_) value set at 4 and the p-value at 0.05.

### 2.5 qRT-PCR

qRT-PCR was conducted using the BioRad CFX Connect™ Real-Time System (CFX Connect™; Bio-Rad) and iTaq Universal SYBR^®^ Green Supermix (CFX Connect™; Bio-Rad).

Based on the DEG data analysis using a two-fold change threshold, laccase-related genes showing increased expression were selected for qRT-PCR validation. Specifically, *Laccase* (*LACC2*) from *Pleurotus ostreatus*, *Laccase-7* (AGABI2DRAFT_239300) and *Laccase-9* (AGABI2DRAFT_194714) from *Agaricus bisporus*, and *Laccase* (C8R40DRAFT_642440) from *Lentinula edodes* were chosen for further analysis. Primers for the selected gene were designed by querying the NCBI (National Center for Biotechnology Information) database using the gene symbol from ExDEGA. The designed primers were listed in **Table 2**. Actin genes were used as internal controls for normalization in *P. ostreatus* (PO_2*), A. bisporus* (AB_3), and *L. edodes* (LE_2).

**Table 2:** Detailed information on primers used for qRT-PCR analysis.

Name	Fold change T/Con^a^	Squence (5’-3’)	Tm^b^(°C)	Product size (bp)
PO_1^*^	186.86	F: GAAGCTGGTCTCGCTGTTGTC R: GTTCGCCCTCGTTGACTTCA	F: 61.5°C R: 60.6°C	58
PO_2	-	F: CGCATGCAGAAGGAGTTGAC R: GCGATGAACAATAGCAGGGC	F: 59.3°C R: 59.3°C	183
AB_1	7.448	F:TCTCGTGCCCTTCCTGTTTC R:TGATAGTACCCTTCGGCCCA	F: 59.3°C R: 59.3°C	149
AB_2	7.311	F:CCCAGTGGAATGCGTTCTCT R:GGGCGTAGGTAAGATGGACG	F: 60.0°C R: 59.9°C	107
AB_3	-	F:TACCCGATCGAACACGGTAT R:GCCACTCGCAATTCATTGTA	F: 57.3°C R: 55.2°C	86
LE_1	5.437	F:CCTCACCATCATCGAGGTCG R:TATTGGTCGGTTGGCGTTGA	F: 59.9°C R: 59.9°C	112
LE_2	–	F:GTGTTACCCATACCGTTCCC R:ATCGGTCAAATCACGACCAG	F: 59.3°C R: 57.3°C	89

^*^PO_1, *P. ostreatus* laccase (poxa3, AJ344434.2); PO_2, *P. ostreatus* β-actin (AY772706.1); AB_1, *A. bisporus* laccase-7 (XM_006460562.1); AB_2, *A. bisporus* laccase-9 (XM_006464104.1); AB_3, *A. bisporus* actin-1 (XM_006460331.1); LE_1, *L. edodes* laccase (XM_046235749.1); LE_2, *L. edodes* actin-1 (XM_046231470.1).

a T/Con, Treated mushroom/Untreated mushroom.

b Tm, melting temperature.

qRT-PCR was performed with an initial denaturation step at 95°C for 30 seconds, followed by 39 cycles of denaturation at 95°C for 3 seconds and annealing/extension at 54°C for 20 seconds. The relative expression levels of each gene were calculated using the 2^-⊿⊿Ct^ method based on the Ct values and average Ct values obtained (Livak and Schmittgen, 2001). Each qRT-PCR reaction was conducted in technical triplicate.

## 3 Results

### 3.1 Effects of the pathogen on the fruiting bodies

#### 3.1.1 Disease symptoms and extent of browning

**Figure 1:**
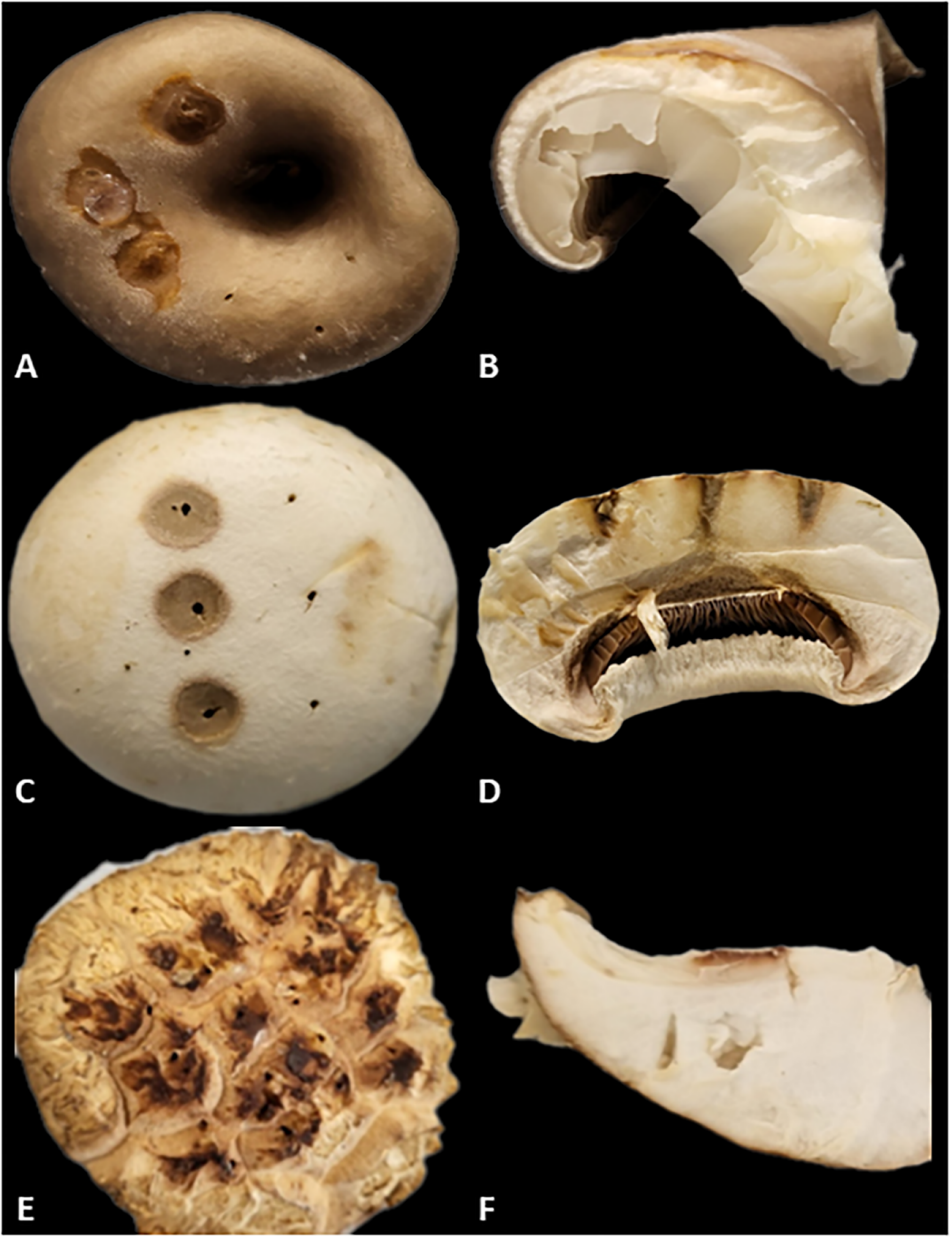
Analysis of surface and cross-sectional browning in mushrooms inoculated with *P. tolaasii***. (A)** Pileus of *P. ostreatus* (surface). **(B)** Pileus of *P. ostreatus* (cross section). **(C)** Pileus of *A. bisporus* (surface). **(D)** Pileus of *A. bisporus* (cross section). **(E)** Pileus of *L. edodes* (surface). **(F)** Pileus of *L. edodes* (cross section).

After inoculation, the disease symptoms observed on the fruiting bodies caused by *P. tolaasii* are illustrated in **Figure 1**. As indicated in the study, the area where the *P. tolaasii* colony segment was applied turned brown, and lesions developed on the surface and cross section of the pileus, accompanied by the presence of sticky mucus and a foul odor (Jeon et al., 2003; Kim et al., 2017; Storey et al., 2020; Liu et al., 2016). Browning was observed in *P. ostreatus*, *A. bisporus*, and *L. edodes* although the extent of browning varied among the species. Despite creating a 1cm wound with a syringe needle of consistent thickness, the differences observed are attributed to variations in the density of mycelial tissues within each fruiting body (Cho et al., 2000).

**Figure 2:**
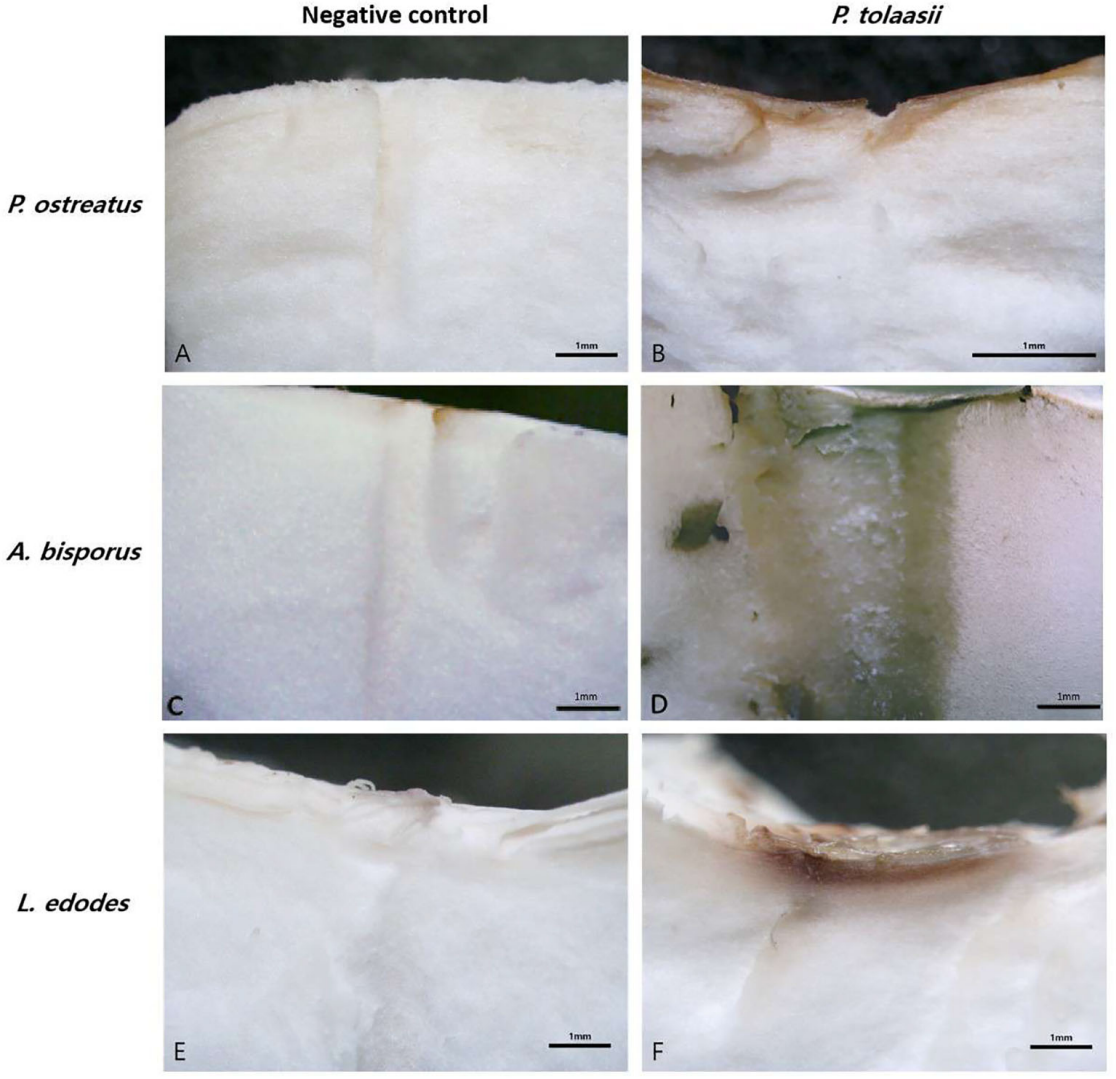
Analysis using a stereoscopic microscope after *P. tolaasii* inoculation. **(A)** Treated LB Agar. **(B)** Treated *P. tolaasii*. **(C)** Treated LB Agar. **(D)** Treated *P. tolaasii*. **(E)** Treated LB Agar. **(F)** Treated *P. tolaasii*.

To confirm that *P. tolaasii* induces browning and tissue softening in mushroom fruiting bodies, a comparison was made between a treatment involving a segment of a *P. tolaasii* colony and a treatment using an LB agar segment. In the LB agar treatment, a slight browning reaction was observed on the surface of the fruiting body, and its cross-section was examined under a dissecting microscope (**Figure 2**).

As a result, the LB agar treatment induced browning exclusively on the directly stimulated surface, which is likely attributable to mechanical wounding caused by the agar plug. In contrast, the *P. tolaasii* treatment caused browning not only at the site of contact with the colony segment but also within the cavity created by the syringe needle. These observations indicate that the brown lesions formed inside the fruiting bodies were specifically induced by *P. tolaasii*.

Thus, transcriptome analysis was conducted to investigate the causes of the morphological changes induced in mushrooms by *P. tolaasii*.

### 3.2 Gene expression patterns induced by *P. tolaasii*

#### 3.2.1 Comparative analysis of gene expression

To enable an unbiased comparison of differentially expressed genes across species, orthologous relationships among *P. ostreatus*, *A. bisporus*, and *L. edodes* were established using OrthoFinder. Differential expression results were interpreted at the orthogroup level, and overlap analysis was performed based on shared orthologs rather than direct gene ID matching.

In the DEG results comparing fruiting bodies with induced disease symptoms to those without, a total of 12,457 transcripts were identified for *P. ostreatus*. Applying criteria of a fold change of at least 2, normalized data (log_2_) of at least 4, and a p-value of 0.05 or less, 785 genes were found to be significantly upregulated, while 886 genes were significantly downregulated. Similarly, for *A. bisporus*, a total of 10,601 transcripts were identified, and using the same criteria, 557 genes were significantly upregulated and 583 genes were significantly downregulated. In the case of *L. edodes*, a total of 14,389 transcripts were identified, and applying the same criteria, 1,098 genes were significantly upregulated and 1,003 genes were significantly downregulated.

To verify the functions of the expressed genes, hypothetical proteins were excluded from the analysis. In *P. ostreatus*, 47 upregulated genes and 75 downregulated genes were confirmed; in *A. bisporus*, 50 and 28, respectively; and in *L. edodes*, 479 and 381, respectively (**Table 3**).

**Table 3:** The number of genes as a result of DEG (Differentially Expressed Gene).

Name	Total	Up-regulation		Down-regulation	
		IHG*	EHG*	IHG*	EHG*
*P. ostreatus*	12,457	785	47	886	75
*A. bisporus*	10,601	557	50	583	28
*L. edodes*	14,389	1,098	479	1,003	381

*IHG, Inclusion Hypothetical protein coding Genes; EHG, Elimination of Hypothetical proteins coding Genes.

Significant DEGs identified in *P. ostreatus*, *A. bisporus*, and *L. edodes* are shown here. A fold change (FC) value of 1 or greater indicates increased expression, while an FC value of less than 1 indicates decreased expression. Differentially expressed genes were selected based on the criteria of log_2_ fold change ≥ 2.00, normalized expression ≥ 4.00, and p-value ≤ 0.05. This study has the limitation that only one biological replicate was available per condition; therefore, gene-level p-values and FDR-adjusted values could not be calculated, and the identified DEGs should be interpreted as exploratory.

In *P. ostreatus*, *A. bisporus*, and *L. edodes*, genes showing significant up- or down-regulation were identified, and the detailed lists of differentially expressed genes for each species are summarized in **Tables 4**–**6**.

**Table 4:** Differentially expressed gene list of fruit body for *P. ostreatus*.

Gene ID	FC^a^	Annotation
	PO/Con^b^	product
LACC2	186.860	Laccase
DyP4	156.648	DyP-type peroxidase
LACC6	151.870	Laccase
PHOH-1	20.061	PHO4 superfamily
PLEOSDRAFT_1090819	4.424	Catalase
MnP4	2.148	MnP-short, short manganese peroxidase
HTP1	2.136	Heme-thiolate peroxidase
PLEOSDRAFT_1076087	2.050	Serine /threonine protein kinase RIM15
PLEOSDRAFT_1065866	1.550	putative Tco5 histidine kinase
PLEOSDRAFT_1055491	0.359	Glycoside hydrolase family 17 protein
PLEOSDRAFT_1104273	0.324	Glycoside hydrolase family 76 protein
PLEOSDRAFT_61779	0.293	Glycoside hydrolase family 2 protein
PLEOSDRAFT_1053206	0.142	Glycoside hydrolase family 47 protein
PLEOSDRAFT_31568	0.097	Hydrophobin modified 4
Hydph13	0.084	ClassIhydrophobin superfamily
PLEOSDRAFT_20823	0.060	Glycoside hydrolase family 13 protein
PLEOSDRAFT_159371	0.049	Glycoside hydrolase family 16 protein
Hydph18	0.015	ClassIhydrophobin superfamily
PLEOSDRAFT_1044883	0.007	Putative classIhydrophobin superfamily

a FC, Fold change.

b PO, Treated *P. ostreatus* (*P. tolaasii*); Con, Untreated *P. ostreatus*.

**Table 5:** Differentially expressed gene list of fruit body for *A. bisporus*.

Gene ID	FC^a^	Annotation product
	AB/Con^b^	
AGABI2DRAFT_120936	46.459	Cytochrome P450
AGABI2DRAFT_191532	18.378	Tyrosinase
AGABI2DRAFT_194888	17.205	ABL galactose binding lectin
AGABI2DRAFT_230189	15.638	Pyranose dehydrogenase
AGABI2DRAFT_75698	9.187	XCL-like lectin
AGABI2DRAFT_239300	7.448	Laccase-7
AGABI2DRAFT_194714	7.311	Laccase-9 precursor
AGABI2DRAFT_194055	5.404	Polyphenol oxidase
AGABI2DRAFT_192776	3.936	Phenylalanine ammonia-lyase
AGABI2DRAFT_239410	3.569	Catalase
AGABI2DRAFT_196962	3.212	Exo-1,3-beta-glucanase
AGABI2DRAFT_192690	2.852	Phenylalanine ammonia-lyase
AGABI2DRAFT_184993	2.073	Laccase-12
AGABI2DRAFT_64285	2.006	Glutamate 5-kinase
AGABI2DRAFT_143539	1.026	Tco5 type IB histidine kinase sensor protein
AGABI2DRAFT_211997	0.439	Glycoside hydrolase family 13 protein
AGABI2DRAFT_143465	0.292	Hydrophobin
AGABI2DRAFT_133693	0.287	Hydrophobin-1
AGABI2DRAFT_191083	0.020	B-(1-6) glucan synthase

a FC, Fold change.

b AB, Treated *A. bisporus* (*P. tolaasii*); Con, Untreated *A. bisporus*.

**Table 6:** Differentially expressed gene list of fruit body for *L. edodes*.

Gene ID	FC^a^	Annotation product
	LE/Con^b^	
C8R40DRAFT_1099187	484.215	Kinase-like domain-containing protein
C8R40DRAFT_1172906	157.182	Delta-endotoxin CytB
C8R40DRAFT_1164647	95.842	Cytochrome P450
C8R40DRAFT_1045742	82.572	Concanavalin A-like lectin/glucanase domain-containing protein
C8R40DRAFT_1108749	31.755	Aldo /keto reductase
C8R40DRAFT_1036880	26.573	Alcohol dehydrogenase
C8R40DRAFT_1158968	18.493	Putative MAP kinase
C8R40DRAFT_1177332	7.336	Heme peroxidase
C8R40DRAFT_1176007	7.216	Exo-beta-1,3-glucanase
C8R40DRAFT_1163247	7.051	Cyclophilin
C8R40DRAFT_642440	5.437	Laccase
C8R40DRAFT_1053306	4.929	Pleiotropic drug resistance ABC transporter
C8R40DRAFT_1043910	4.907	ABC protein
C8R40DRAFT_774955	4.129	2 beta-glucanase
C8R40DRAFT_28479	1.113	histidine kinase
C8R40DRAFT_1051083	0.445	Glycoside hydrolase family 2 protein
C8R40DRAFT_1068220	0.444	Glycoside hydrolase family 76 protein
C8R40DRAFT_1239604	0.430	1,3-beta-glucan synthase
C8R40DRAFT_1204419	0.323	Glycoside hydrolase family 47 protein
C8R40DRAFT_1130231	0.290	Glycoside hydrolase family 13 protein
C8R40DRAFT_610256	0.221	Glycoside hydrolase family 16 protein
C8R40DRAFT_1109817	0.208	Glycoside hydrolase family 17 protein
C8R40DRAFT_775537	0.146	2 beta-glucan
C8R40DRAFT_1086773	0.028	Hydrophobin 1
C8R40DRAFT_555748	0.016	Fungal hydrophobin- domain-containing protein

a FC, Fold change.

b LE, Treated *L. edodes* (*P. tolaasii*); Con, Untreated *L. edodes*.

**Figure 3:**
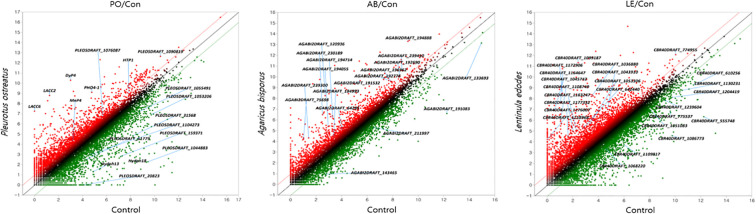
The DEG scatter plot analysis of *P. ostreatus, A. bisporus* and *L. edodes*. PO/Con, Treated *P. ostreatus*/untreated *P. ostreatus*; AB/Con, Treated *A. bisporus*/untreated *A. bisporus*; LE/Con, Treated L. *edodes*/untreated *L. edodes*.

Based on the criteria of a fold change of at least 2, normalized data (log_2_) of at least 4, and a p-value of 0.05 or less, genes exhibiting significant changes in expression were plotted on a DEG scatter plot. Genes with increased expression were represented in red, while those with decreased expression were depicted in green. In P. ostreatus, 8 genes were upregulated and 9 genes were downregulated; in A. bisporus, 14 genes were upregulated and 4 genes were downregulated; and in L. edodes, 14 genes were upregulated and 10 genes were downregulated (**Figure 3**).

Among these, genes selected for the scatter plot were those that not only met the statistical criteria but were also considered biologically meaningful in relation to pathogen defense mechanisms.

Among the commonly expressed orthologous genes across the three species, those related to defense mechanisms exhibited similar expression patterns, which were consistently observed across species. These findings suggested the possibility of a shared defense response mechanism among edible mushrooms against *P. tolaasii* infection.

The catalase gene was commonly upregulated in both *P. ostreatus* and *A. bisporus*. In addition, genes related to cytochrome P450, β-glucanase, and lectin were commonly upregulated in *A. bisporus* and *L. edodes*. In all three species, *P. ostreatus*, *A. bisporus*, and *L. edodes*, genes related to laccase and histidine kinase were commonly upregulated. In addition, glycoside hydrolase family 2, 47, 76, 16, and 17 proteins were downregulated in *P. ostreatus* and *L. edodes*, while the β-glucan synthase-related gene was downregulated in both *A. bisporus* and *L. edodes*. Furthermore, hydrophobin and glycoside hydrolase family 13 protein were commonly downregulated in all three species.

**Figure 4:**
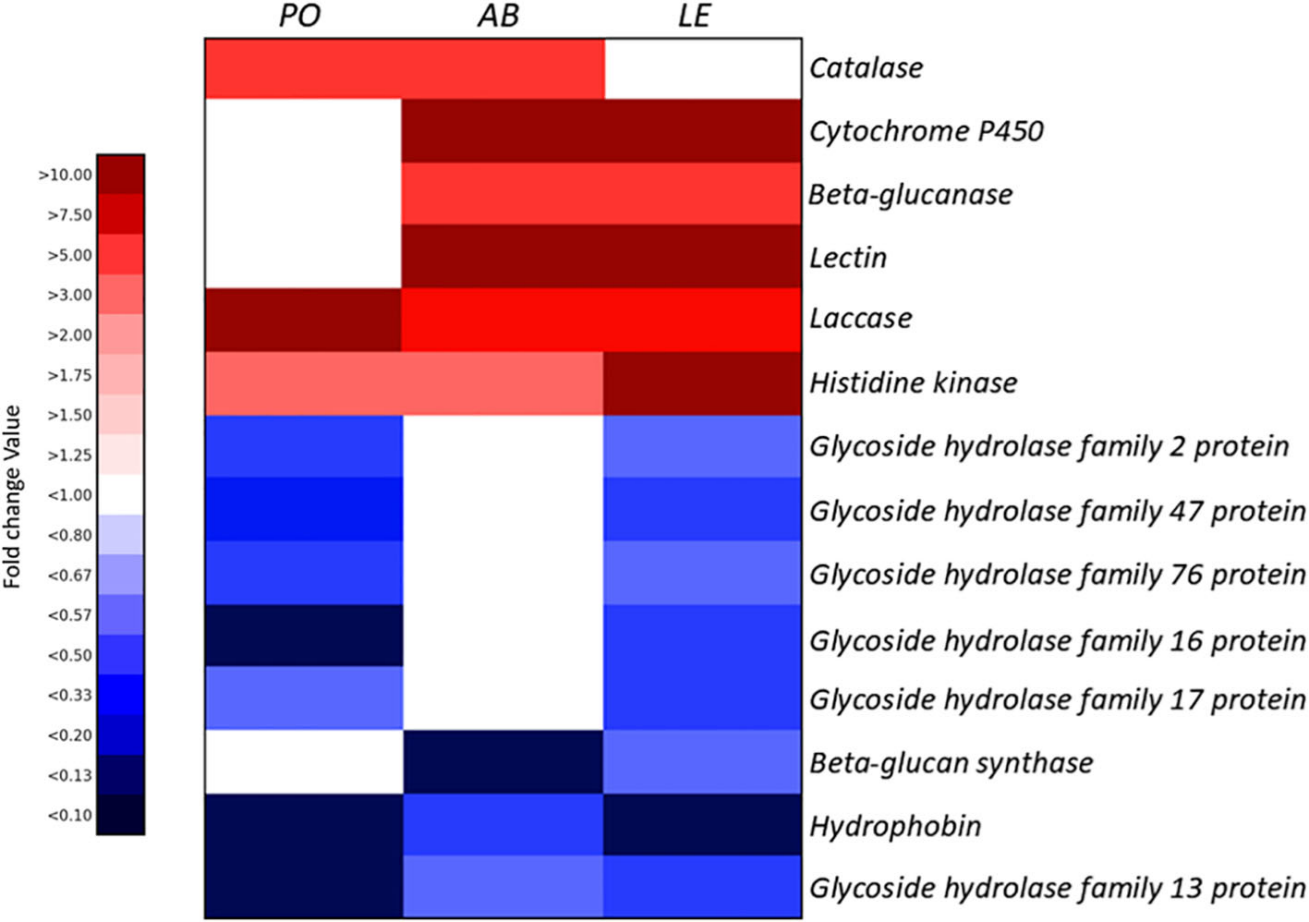
The heatmap of common genes for *P. ostreatus*, *A. bisporus*, and *L. edodes*. PO, *P. ostreatus*; AB, *A. bisporus*; LE, *L. edodes*.

**Figure 5:**
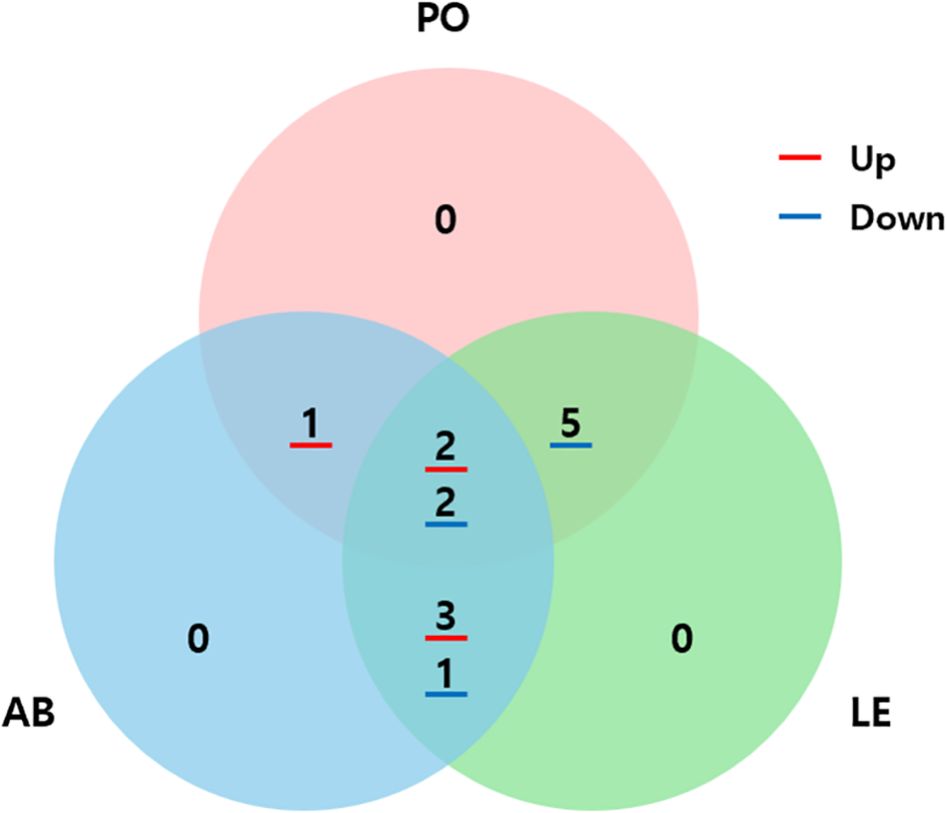
Venn diagram analysis of common genes in *P. ostreatus*, *A. bisporus* and *L. edodes* in response to *P. tolaasii*. PO, *P. ostreatus*; AB, *A. bisporus*; LE, *L. edodes*.

These gene expression patterns were visualized using the heatmap in **Figure 4**, and the commonly expressed DEGs among the three species were illustrated in the Venn diagram shown in **Figure 5**. All commonly expressed defense-related genes were confirmed as orthologs across the three species by OrthoFinder analysis. For instance, laccase genes were assigned to a shared orthogroup across *P. ostreatus*, *A. bisporus*, and *L. edodes*, and histidine kinase genes were likewise clustered into a common orthogroup. Detailed orthogroup assignments for all other gene families are provided in Supplementary Data 2, as the complete list is too extensive to present in the main text.

#### 3.2.2 qRT-PCR

Among the laccase genes identified as orthologous across *P. ostreatus*, *A. bisporus*, and *L. edodes* based on OrthoFinder analysis, Laccase7 and Laccase9 from *A. bisporus*, LACC2 from *P. ostreatus*, and a laccase gene from *L. edodes* were selected for qRT-PCR validation.

**Figure 6:**
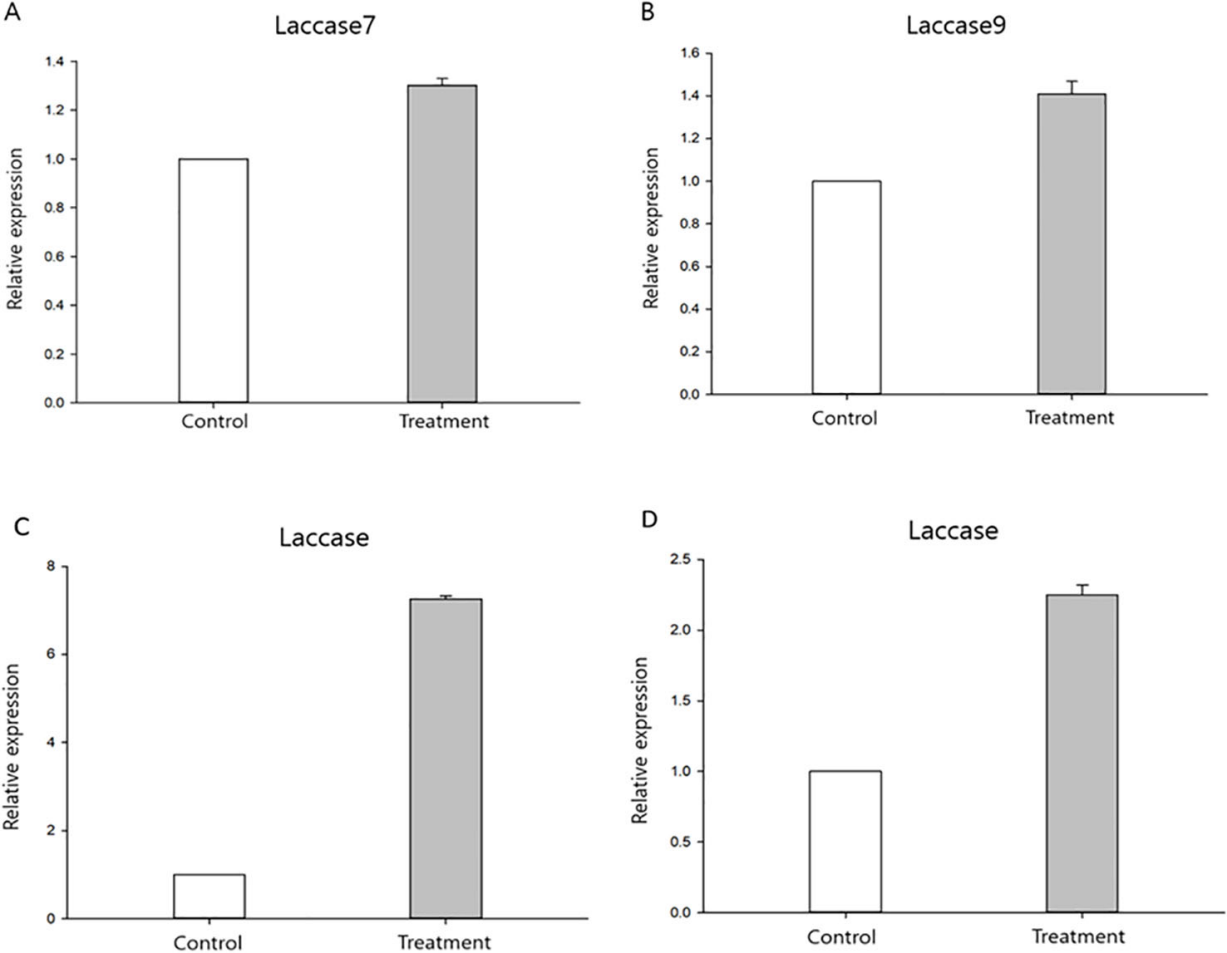
qRT-PCR results normalized using ^⊿⊿^ct method. ^*^Control, Untreated mushroom; Treated, Treated *P. tolaasii* mushroom. **(A)** *A. bisporus* AGABI2DRAFT_239300. **(B)** *A. bisporus AGABI2DRAFT_194714*. **(C)** *P. ostreatus LACC2*. **(D)** *L. edodes* C8R40DRAFT_642440. Data represent mean ± standard deviation (n = 3 biological replicates). Statistical analysis of ΔCt values showed a significant difference in *P. ostreatus LACC2* (p < 0.01), while no significant differences were detected in *A. bisporus* Laccase7/9 or *L. edodes* Laccase (p > 0.05).

The results showed overall trends consistent with the DEG analysis (**Figure 6**), although statistical significance was only confirmed for *P. ostreatus LACC2* (p = 0.0065). In contrast, no statistically significant differences were detected for *A. bisporus* Laccase7 (p = 0.959), *A. bisporus* Laccase9 (p = 0.258), and *L. edodes* Laccase (p = 0.096). Actin genes used as internal controls were PO_2 for *P. ostreatus*, AB_3 for *A. bisporus*, and LE_2 for *L. edodes*. However, as agarose gel electrophoresis and melting curve analyses for primer validation were not performed, these qRT-PCR results should be interpreted with caution and primarily in the context of their consistency with the RNA-seq data.

## 4 Discussion

Currently, research on bacterial brown blotch disease caused by *P. tolaasii* has primarily focused on the development of eco-friendly inhibitors utilizing the pathogen’s toxins (Lee et al., 2014; Yun et al., 2017; Sullivan, 2015). However, studies investigating the resistance of edible mushrooms to *P. tolaasii* infection have been limited. Therefore, in this study, we induced browning through artificial inoculation with *P. tolaasii* and analyzed the commonly expressed genes involved in the defense mechanisms and browning responses in *P. ostreatus*, *A. bisporus*, and *L. edodes*.

According to the results of this study, browning was observed in *P. ostreatus*, *A. bisporus*, and *L. edodes* following artificial inoculation with *P. tolaasii*. This phenomenon was likely associated with the oxidation of phenolic compounds mediated by polyphenol oxidase (PPO), which is known as one of the pathways that can lead to melanin production (Sullivan, 2015; Janusz et al., 2020). In addition, laccase, a type of PPO, is known to be involved in the melanin biosynthesis pathway by oxidizing phenolic substances through a multi-copper oxidase that contained a copper atom at the center of its active site (Meitil et al., 2025). Fungal melanin, a secondary metabolite consisting of phenolic and indolic monomers, contributes to morphogenesis, pathogenicity, and energy conversion, and protects cellular viability by mitigating environmental stress (Toledo et al., 2017). In fungi, melanogenesis mediated by PPOs such as tyrosinase and laccase is restricted to specific developmental stages and wound-associated conditions, with melanin deposited on the cell wall surface where it is cross-linked with polysaccharides, thereby enhancing resistance to hydrolytic enzymes and environmental stress (Bell and Wheeler, 1986; McMahon et al., 2007; Eisenman and Casadevall, 2012; Solano, 2014).

Moreover, laccase is activated not only under biotic stresses, such as pathogen infection, but also under abiotic stresses, including oxidative stress, heavy metals, and environmental changes. Research has demonstrated that laccase plays a crucial antioxidant role by removing reactive oxygen species (ROS), which was essential for fungal survival. Additionally, it exhibits a strong capacity for detoxifying exogenous toxic substances (Zhong et al., 2019). In fact, when *Morchella sextelata* was infected by the pathogenic fungus *Paecilomyces penicillatus*, the pathogen was found to synthesize and secrete phenolic toxins into the host. In response, *M. sextelata* appeared to activate the expression of *laccase* to detoxify these phenolic compounds (Yu et al., 2021).

Although the exact chemical structure of the toxin has not yet been elucidated, this defense mechanism was similar to that observed in *Agaricus bisporus*. In that case, the activity of *laccase-2* was found to enhance resistance against the toxin produced by the pathogen *Trichoderma aggressivum*, specifically 3,4-dihydro-8-hydroxy-3-methyl isocoumarin, which contains a phenolic hydroxyl group and a lactone ring (Sjaarda et al., 2015).

In the present study, several laccase genes were chosen for qRT-PCR validation to examine their relationship with RNA-Seq data and defense against disease. Specifically, *LACC2* (the most highly expressed laccase) was selected from *P. ostreatus*; laccase-7 (AGABI2DRAFT_239300) and the laccase-9 precursor (AGABI2DRAFT_194714) were selected from *A. bisporus*; and laccase (C8R40DRAFT_642440) was selected from *L. edodes*. As illustrated in **Figure 6**, the expression of laccase-related genes increased in all mushroom species following infection with *P. tolaasii*.

These findings suggested that the increased expression of laccase genes might be functionally associated with defense responses against pathogens in each species. However, laccase genes are known to perform diverse physiological functions depending on the species. In *P. ostreatus*, the PoLac2 gene selected for qRT-PCR analysis has been reported to play a major role in lignin degradation, and its overexpression has been shown to enhance ligninolytic activity (Jiao et al., 2018). In *A. bisporus*, laccase-7 and laccase-9 have been suggested to be involved in stress responses and secondary metabolism, including functions related to disease resistance and lignin modification (Sjaarda et al., 2015). In *L. edodes*, intracellular laccase in the fruiting body has been identified as directly contributing to melanin biosynthesis, a function that may be associated with both pigmentation and pathogen defense (Nagai et al., 2003). Overall, these previous studies indicate that the laccase genes selected for qRT-PCR analysis in this study are involved in various physiological processes such as lignin degradation, secondary metabolism, and melanin biosynthesis in each species. Their increased expression following pathogen inoculation is therefore considered to contribute to species-specific defense strategies. Although it remains unclear whether tolaasin is a direct substrate of laccase, a study by Sjaarda et al. (2015) demonstrated the potential role of laccase activity in detoxifying pathogenic toxins in *A. bisporus*. In confrontation assays between *Schizophyllum commune* and *Trichoderma harzianum* or *Trichoderma aggressivum*, a distinct dark line was formed in the interaction zone, accompanied by the induction of defense-related proteins, including secreted lectins and thaumatin-like proteins (Beijen et al., 2024). In particular, treatment with toxic extracts of *Trichoderma aggressivum* induced the expression of the lcc2 gene, suggesting that laccase may serve a defensive role under stress conditions (Sjaarda et al., 2015).

In addition to laccase, the differential expression analysis of genes revealed the significance of the two-component system (TCS). TCS is a crucial signaling mechanism that allowed bacteria and certain eukaryotes to adapt to environmental changes (Alvarez and Georgellis, 2023). Among the signal transduction proteins, histidine kinase (HK) plays a vital role by detecting environmental signals, undergoing autophosphorylation, and subsequently transferring the phosphate group to the response regulator (RR) to modulate gene expression (Catlett et al., 2003; Lavín et al., 2010).

Concerning histidine kinase-related genes, increased expression was observed in *P. ostreatus* for the putative Tco5 histidine kinase (PLEOSDRAFT_1065866), in *A. bisporus* for the Tco5 type IB histidine kinase sensor protein (AGABI2DRAFT_143539), and in *L. edodes* for histidine kinase (C8R40DRAFT_28479). Consistent with previous reports indicating that HK in fungi was involved in regulating the expression of oxidases such as laccase (Motoyama et al., 2008; Cripps et al., 1990), the present study suggested that the increased expression of HK in the fruiting bodies of *P. ostreatus*, *A. bisporus*, and *L. edodes* infected with *P. tolaasii* was associated with the regulation of laccase expression.

Hydrophobins are small, secreted cysteine-rich proteins that formed hydrophobic coatings on the fungal cell wall (So and Kim, 2017; Agwora et al., 2025; Rojas-Osnaya et al., 2024). The expression of hydrophobins is regulated during mycelial formation, primordia development, and fruiting body formation, thereby playing a crucial role in the environmental adaptation and growth of fungi (Xu et al., 2021).

Glycoside hydrolase family 13 (GH13) proteins belong to the α-amylase family and were known for their ability to hydrolyze glycosidic bonds, thereby facilitating the breakdown of carbohydrates (Cantarel et al., 2009; Zhao et al., 2014). α-Amylases, such as Amy1p in Histoplasma capsulatum and AmyD in *Aspergillus niger*, play a crucial role in the synthesis of α-glucan within the fungal cell wall, which was essential for mycelial growth and the maintenance of structural stability (Van et al., 2007).

Based on these previous studies, it was presumed that artificial inoculation with *P. tolaasii* induced tissue degradation and impaired cellular structure and function, leading to the disruption of the cell wall, which played a critical role in maintaining cell morphology, and this appeared to alter the expression patterns of genes related to cell wall composition, such as hydrophobin and GH13 family genes.

In this study, *P. tolaasii* was artificially inoculated into *P. ostreatus*, *A. bisporus*, and *L. edodes* to induce browning, and qRT-PCR was performed to focus on the associated laccase enzymes. Although various laccase family genes were present in each mushroom species (Sjaarda et al., 2015; Jiao et al., 2018; Yan et al., 2019). This study aimed to analyze the major laccase genes related to defense mechanisms. In the future, comparing gene expression between artificial inoculation and the natural occurrence of *P. tolaasii* will allow for a clearer elucidation of expression patterns. The browning-related genes identified in this study will serve as important foundational data for future research on the defense mechanisms of *P. ostreatus*, *A. bisporus*, and *L. edodes* against *P. tolaasii* infection.

Therefore, based on the results of this study, analyzing the accumulation of reactive oxygen species (ROS) and changes in *laccase* expression in fruiting bodies infected with *P. tolaasii* will help clarify whether browning acts as a defense mechanism against pathogen infection. This approach is expected to contribute to a better understanding of the molecular mechanisms underlying oxidative stress responses and disease resistance in mushrooms, and will ultimately aid in addressing issues in mushroom cultivation and improving mushroom quality by controlling disease development.

## Data Availability

The datasets presented in this study can be found in online repositories. The names of the repository/repositories and accession number(s) can be found in the article/**Supplementary Material**.
